# Sanguinarine, identified as a natural alkaloid LSD1 inhibitor, suppresses lung cancer cell growth and migration

**DOI:** 10.22038/IJBMS.2022.62541.13851

**Published:** 2022-06

**Authors:** Ting-ting Qin, Zhong-hua Li, Li-xin Li, Kun Du, Ji-ge Yang, Zhen-qiang Zhang, Xiang-xiang Wu, Jin-lian Ma

**Affiliations:** 1 Academy of Chinese Medical Sciences, Henan University of Chinese Medicine, Zhengzhou 450046, Henan Province, China; 2 School of Pharmacy, Henan University of Chinese Medicine, Zhengzhou 450046, Henan province, China

**Keywords:** Antiproliferation, LSD1 inhibitor, Migration, NSCLC, Sanguinarine

## Abstract

**Objective(s):**

Lysine-specific demethylase1 (LSD1), an important class of histone demethylases, plays a crucial role in regulation of mammalian biology. The up-regulated LSD1 expression was frequently associated with progress and oncogenesis of multiple human cancers, including non-small cell lung cancer (NSCLC). Therefore, inhibition of LSD1 may provide an attractive strategy for cancer treatment. We investigated the effect of sanguinarine against lung cancer cells as a natural alkaloid LSD1 inhibitor.

**Materials and Methods:**

The inhibition properties of sanguinarine to the recombinant LSD1 were evaluated by a fluorescence-based method. Subsequently, assays such as viability, apoptosis, clonogenicity, wound healing, and transwell were performed on H1299 and H1975 cells after treatment with sanguinarine.

**Results:**

Upon screening our in-house natural chemical library toward LSD1, we found that sanguinarine possessed a potent inhibitory effect against LSD1 with the IC50 value of 0.4 μM in a reversible manner. Molecular docking simulation suggested that sanguinarine may inactivate LSD1 by inserting into the binding pocket of LSD1 to compete with the FAD site. In H1299 and H1975 cells, sanguinarine inhibited the demethylation of LSD1, validating its cellular activity against the enzyme. Further studies showed that sanguinarine exhibited a strong capacity to suppress colony formation, inhibit migration and invasion, as well as induce apoptosis of H1299 and H1975 cells.

**Conclusion:**

Our findings present a new chemical scaffold for LSD1 inhibitors, and also provide new insight into the anti-NSCLC action of sanguinarine.

## Introduction

Lung cancer has long remained the leading cause of cancer-related death worldwide over the past decades. As the most common subtype of lung cancer, non-small-cell lung cancer (NSCLC) accounts for about 85% of lung cancer diagnoses (1). Despite important advancements in chemotherapy, molecular targeting therapy, and biological mechanisms, the prognosis for NSCLC remains grim. The lack of biomarkers for early diagnosis and effect target for therapy is still the most significant challenge for NSCLC. Therefore, it is of great significance to find specific targets for lung cancer and develop new drugs for NSCLC.

In recent years, epigenetic abnormalities have been demonstrated to be involved in all the stages of cancer development, to promote cancer initiation and progression. Histone lysine-specific demethylase 1 (LSD1) is considered to be a crucial epigenetic regulator with the ability to demethylate mono-, di-methylated K4, and K9 of histone 3 in flavin adenine dinucleotide (FAD)-dependent manner, resulting in transcriptional repression and activation. In addition, LSD1 was identified to demethylate nonhistone substrates (e.g., p53, E2F transcription factor 1, and DNA methyltransferases) and further regulated their downstream cellular function (2-4). It has been reported that the overexpression of LSD1 is strongly associated with the progress of malignant cancers, including gastric (5), breast (6), prostate (7), colon (8), lung cancers (9), and others (10).

Several lines of studies have shown that LSD1 can promote the occurrence and development of cancer by regulating the processes of lipid metabolism, epithelial-mesenchymal transition (EMT), apoptosis, and autophagy of cancer cells (5, 11-15). Specifically, LSD1 is frequently highly expressed in lung cancer and tightly associated with the adverse prognosis of lung cancer (9, 16-17). LSD1 is capable to promote SCLC progression through transcriptional silencing of the tumor suppressor Rest (18). Recent studies reported that LSD1 can up-regulate SEPT6 to activate the TGF-β1 pathway and subsequently promote the metastasis of NSCLC (19). Inhibition of LSD1 by genetic depletion or pharmacological intervention with LSD1 inhibitor can cause the reduction of cancer cell proliferation and induction of apoptosis. Therefore, LSD1 has become an attractive molecule target for developing specific inhibitors as anticancer agents. 

To date, a large number of LSD1 inhibitors with varieties of structures have been developed, and several synthetic inhibitors of LSD1 such as TCP, ORY-1001, GSK2879552, and IMG-7289, are currently under investigation in clinical trials for treatment of hematologic malignancies, lung cancers, etc. In addition, as a rich source of bioactive molecules, several natural products such as mangostin, catechol, and baicalin have been successively identified as novel LSD1 inhibitors with moderate inhibitory activities (20-23). The current reported natural LSD1 inhibitors mainly belong to the flavonoid structural set, and search for other natural skeletons such as alkaloid remains of interest.

Sanguinarine is a natural alkaloid featured with benzophenanthridine polycycle skeleton, which is mainly derived from poppy fumaria species. Sanguinarine has a wide range of pharmacological activities, such as anti-microbial (24, 25), anti-tumor (26), anti-inflammatory (27), and anti-angiogenesis (28). Following our previous work on the identification of novel LSD1 inhibitors, we screened our in-house natural chemical library toward LSD1 and found that sanguinarine exhibited good inhibitory activity against LSD1. Herein, we report our efforts toward the identification of sanguinarine as a potent, reversible, and cellular active LSD1 inhibitor, as well as its anti-NSCLC activity.

## Materials and Methods

***LSD1 inhibitory evaluation***

The assay for LSD1 inhibition was performed according to our previously reported method (29). The prepared plasmid pET-28b-LSD1 was transfected into BL21 (DE)3 and 0.25 mM IPTG was added overnight at 20 ℃ to induce LSD1 overexpression. It was then purified by affinity chromatography and ion-exchange chromatography. Subsequently, the compounds in gradient concentration were incubated with the recombinant and the peptide substrate H3K4me2. Finally, HRP and Amplex Red were added to measure fluorescence at the excitation wavelength of 530 nm and emission wavelength of 590 nm, and the inhibition rate of compounds was calculated. 

***Reversibility analysis***

The reversibility of sanguinarine to LSD1 activity was determined by dilution assay. The LSD1 recombinant was incubated with 400 μM sanguinarine or 50 μM ORY-1001 for 1 hr. Then for the after-dilution group, the enzyme-compound mixture (1.25 μl) was transferred into reaction solution with or without substrate H3K4me2 peptide and coupling reagents to a final volume of 100 μl. The enzyme activity of LSD1 was detected again according to the above method. Meanwhile, for the before-dilution group, the procedure was the same as after-dilution groups. If dilution of the compound concentration significantly changed its inhibitory rate, the compound was a reversible inhibitor. Conversely, an irreversible inhibitor has a similar inhibitory rate after being diluted.

***Molecular docking***

The 3D structure of sanguinarine was built by MOE 2015 and energy-minimized using the force field Amber 10:EHT. The crystal structure of LSD1 (PDB: 2V1D) was prepared by the QuickPrep module using the default parameters, and the FAD pocket was set as the docking site. The default Triangle Matcher placement method was used for docking, and GBVI/WSA dG scoring function to estimate the free energy of binding of sanguinarine from the given pose was used to rank the final pose.

***Cells and cell viability assay***

Human lung cancer cell lines H1975 and A549 were provided by the Cell Bank of the Chinese Academy of Sciences. The human lung cancer cell lines H1299 and H460 were donated by Dr Zheng of Zhengzhou University. Cells were cultured in DMEM/F12 medium or RPMI-1640 medium containing 10% FBS and placed in an incubator with 5% CO2 at 37 °C.

The cells at the exponential stage were placed in a 96-well plate with 5000 cells in each well. After adherence, cells were treated with different concentrations of sanguinarine for 72 hr. Then 20 μl 3-(4, 5-dimethylthiazol-2-yl)-2, 5-diphenyltetrazolium bromide (MTT) solution was added into each well. After 4 hr, the product formazan dissolved in DMSO and was detected at 490 nm using a microplate reader. The data were analyzed using GraphPad Prism 9.0 and SPSS 21.0.

***Clonogenicity assay***

H1975 and H1299 cells were seeded in 6-well plates with 1500 cells in each well. After adherence, the cells were cultured in a medium containing different concentrations of sanguinarine for 48 hr and subsequently were replaced with complete fresh media every two days. After 10–14 days of culture, the colonies were fixed with 4% paraformaldehyde and stained with 0.1% crystal violet solution. After washing twice with PBS, the visible colonies were photographed.

***Wound healing assay***

For the wound healing assay, cells in the logarithmic growth phase were plated in a 6-well plate (150 000 cells/ well). When cells adhered to the wall and reached 70–80% confluence, the cell surface was scratched using a 200 μl pipette tip. The floating cells were washed away with PBS, and the medium with different concentrations of sanguinarine was added to each well and cultured for 48 hr. Finally, the scratch was photographed under an inverted microscope.

***Transwell assay***

For Transwell assay, 15000 cells/Wells were inoculated in the upper chamber of a transwell 24 well plate and cultured in 1% FBS medium with different concentrations of sanguinarine. Medium containing 20% FBS was added to the lower chamber as a chemoattractant. After incubation for 48 hr, cells were washed twice with PBS. The non-invasive cells on the surface of the upper chamber were wiped with cotton swabs, and the cells passing through the membrane were fixed with 4% formaldehyde for 10 min. After 0.1% crystal violet staining for 30 min, pictures were taken under an inverted microscope. Five fields were taken in each chamber and the stained cells were counted.

***Morphological analysis***

The cells in the logarithmic growth phase were plated on a 24-well plate (30,000 cells/ well) with a built-in glass slide to make the cells crawl. The cells were treated with different concentrations of sanguinarine for 48 hr and washed with PBS twice. The cells were immobilized with 4% paraformaldehyde and permeated with 0.5% Triton X-100. Then the nuclei were stained with DAPI, the slide taken out, and placed upside down on the slide. Finally, it was observed and photographed under a fluorescence microscope.

***Western blot***

Cells treated with different concentrations of sanguinarine for 48 hr were collected and then lysed with RIPA lysis or NP-40 lysis buffer containing complete protease inhibitors. The protein concentration was quantified using a BCA commercial kit (Solarbio). Equal amounts of cell lysates were denatured and separated by 8–12% SDS-PAGE and transferred to nitrocellulose (NC) membranes. After being soaked in 5% skim milk for 30 min, the membranes were incubated overnight at 4 °C with primary antibodies. Then the NC membranes were further incubated with secondary antibody for 1 hr at room temperature. The immunoblots were visualized by an enhanced chemiluminescence kit from Thermo Fisher. Images were analyzed by Image J software. Antibodies used were against GAPDH (GOOD HERE, AB-P-R 001), H3 (HUABIO, EM30605), H3K4me2 (HUABIO, R1110-3), H3K9me2 (HUABIO, ET1611-51), CD86 (BOSTER, BM4121), E-Cadherin (HUABIO, ET1607-75), N-Cadherin (HUABIO, ET1607-37), Bax (HUABIO, ER0907), Bcl-2 (HUABIO, ER1082-97).

***Flow cytometry***

The cells were seeded in 6-well plates (250,000 cells/well). After adhering to the wall, cells were treated with different concentrations of sanguinarine for 48 hr and collected. Subsequently, the cells were stained by AnnexinV-FITC/PI Kit from Meilunbio according to the manufacturer’s instructions. Quantification of apoptosis cells was detected by flow cytometry. The early and late apoptotic cells were identified by localization of Annexin V and PI.

***Statistical analyses***

GraphPad Prism 9.0 software was used for data analysis, statistics, and mapping, and SPSS21.0 was used to calculate IC_50_. Data were expressed as mean±SEM. The significance of the difference between different groups was determined with analysis of variance (ANOVA) and Student’s t-test. *(*P*<0.05), **(*P*<0.01), and ***(*P*<0.005) were considered statistically significant compared with the controls.

## Results

***Discovery of sanguinarine as a new natural LSD1 inhibitor***

**Figure 1:**
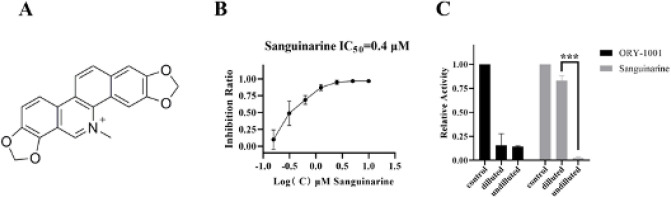
Sanguinarine inhibits LSD1 in a reversible manner at the biochemical level. (A) Structure of sanguinarine; (B) IC_50_ curve of sanguinarine against LSD1; (C) Reversibility of sanguinarine to LSD1 activity was evaluated by dilution assay. Irreversibility inhibitor ORY-1001 was used as a control. Data are the mean ± SEM of three independent experiments. ***(*P*<0.005) vs control

Through screening a natural chemical library toward LSD1, we found that one of these compounds, sanguinarine, exhibited a potent inhibitory effect against LSD1. Meanwhile, in order to exclude the false positive compounds that may react with components other than LSD1 recombinant protein in the screening system, the LSD1 enzyme activity without LSD1 enzyme was tested again for the selected compounds with powerful inhibition. Finally, sanguinarine (Figure 1A) exhibits the potent inhibitory activity of LSD1 with an IC_50_ of 0.4 μM (Figure 1B). To test the reversibility of sanguinarine for LSD1, a dilution assay was performed. The result indicated that dilution of the LSD1/sanguinarine mixture by 80 folds resulted in the recovery of LSD1 activity (Figure 1C), suggesting that sanguinarine may bind and inhibit LSD1 in a reversible manner. ORY-1001 is a known inhibitor of irreversible binding to the LSD1 recombinant protein and can serve as a control (30). As expected, in presence of ORY-1001, LSD1 activity was not recovered after dilution. Our results also confirmed the irreversible inactivation of LSD1 by ORY-1001.

***Molecular docking***

**Figure 2:**
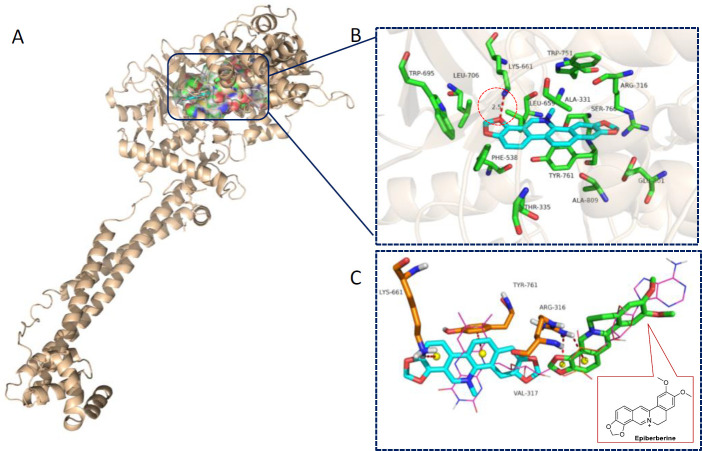
**(**A/B) Predicted binding mode of sanguinarine in the active site of LSD1 (PDB: 2V1D); (C) Overlap of the binding poses of sanguinarine and FAD. For clarity, the residuals are highlighted in green, sanguinarine in cyan, and epiberberine in green

Sanguinarine is featured with a planar and rigid fused-ring aromatic system, and it is highly hydrophobic because of its lack of a polar group. In order to have a preliminary understanding of its inhibitory activity, sanguinarine was docked with the conformation of the LSD1-CoREST complex (PDB: 2V1D) to predict its binding details. As shown in Figure 2A, sanguinarine fit well to the active site of LSD1 and occupied the hydrophobic pocket composed of Tyr761, Leu659, Lys661, Thr335, Ala809, and Val811. The flat polycyclic aromatic core of sanguinarine was predicted to form π-π stacking interactions with Try761 and Leu659. Besides, a dioxole ring formed hydrogen-bonding interaction with Lys661 (2.5 Å, marked in red circle). Moreover, as illustrated in Figure 2C, the result showed that the binding pose of sanguinarine with the lowest energy was well superimposed over the pyrido [3,4-b] quinoxaline moiety of FAD, suggesting that sanguinarine may inhibit LSD1 activity possibly through inserting into the binding cleft of LSD1to compete with FAD. In addition, Li *et al. *(31) reported a set of protoberberine alkaloids as natural LSD1 inhibitors, and epiberberine showed the most potency with the IC_50_ value of 0.14 μM. And the structure-activity relationship revealed that a subtle change in structure led to a significant change in LSD1 inhibition. In view of the structural similarity of sanguinarine with epiberberine, we compared their binding modes in the FAD pocket. As shown in Figure 2C, we found that epiberberine occupied the flexible neck portion of FAD, and sanguinarine was at the rigid bottom, indicating that LSD1 inhibition of this kind of alkaloid structure was quite sensitive to the substituent variation.

***Effects of sanguinarine on LSD1 substrates and CD86 protein expression in NSCLC cells***

**Figure 3:**
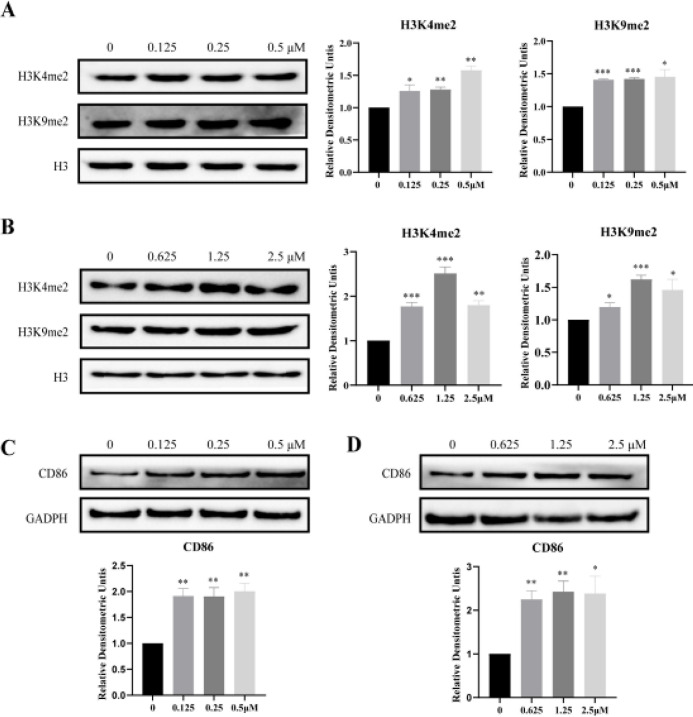
Sanguinarine increased cellular levels of H3K4me2 and H3K9me2 and up-regulated the expression of CD86 in NSCLC cell lines. (A) and (B) Expression of H3K4me2 and H3K9me2 in H1975 and H1299 cells treated with sanguinarine for 48 hr, respectively, with H3 as loading control; (C) and (D) The expression of CD86 was detected, GADPH was used as a loading control. Data are the mean ± SEM of three independent experiments. *P<0.05, **P<0.01, ***P<0.005

To determine whether sanguinarine is a cellular active LSD1 inhibitor, the methylation levels of LSD1 substrates H3K4 and H3K9 were evaluated in NSCLC cell lines H1299 and H1975 treated with sanguinarine. We found that the amounts of H3K4me2 and H3K9me2 were dose-dependently increased after treatment of cells with sanguinarine for 48 hr (Figures 3A, B). In addition, the cell surface marker CD86 has been identified as a sensitive surrogate biomarker of LSD1 activity (32, 33). We further determined the effect of sanguinarine on expression of CD86 in H1299 and H1975 cells. After 48 hr incubation with sanguinarine at indicated concentrations, the protein level of CD86 was markedly elevated (Figure 3C, D). These results suggest that sanguinarine is an effective LSD1 inhibitor at both biochemical and cellular levels.

***Effects of sanguinarine on cell proliferation and colony formation***

**Figure 4.**
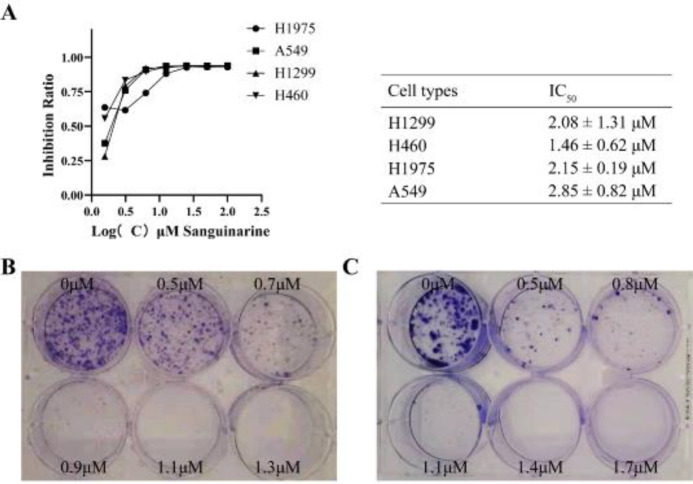
The proliferation of NSCLC cell lines was inhibited by sanguinarine. (A) MTT assay was used to determine the anti-proliferative effects of sanguinarine on H1975, A549, H1299, and H460 cell lines, and the IC_50_ were listed; (B) and (C) Sanguinarine inhibited the clonogenicity of H1975 and H1299; All experiments were repeated at least three times, and *(*P*<0.05), **(*P*<0.01), ***(*P*<0.005) vs control

NSCLC: Non-small cell lung cancer; MTT: 3-(4, 5-dimethylthiazol-2-yl)-2, 5-diphenyltetrazolium bromide

To further investigate whether sanguinarine is a potential candidate for an agent against NSCLC, the cytotoxicity of sanguinarine was evaluated by MTT assay. Four different NSCLC cell lines, namely H1299, H460, H1975, and A549, were treated with sanguinarine for 72 hr. As shown in Figure 4A, sanguinarine exhibited certain antiproliferative activity against these NSCLC cell lines, and IC_50_ was shown respectively. According to the results of the MTT experiment, we selected two cell lines H1975, and H1299 which were more sensitive to sanguinarine for the colony formation experiment to test the ability of sanguinarine to inhibit colony formation. It is shown that sanguinarine significantly reduced the number of cell colonies as concentration increased (Figure 4B, C). Collectively, our results demonstrate that sanguinarine inhibits NSCLC cell proliferation.

***Effects of sanguinarine on apoptosis of NSCLC cells***

**Figure 5.**
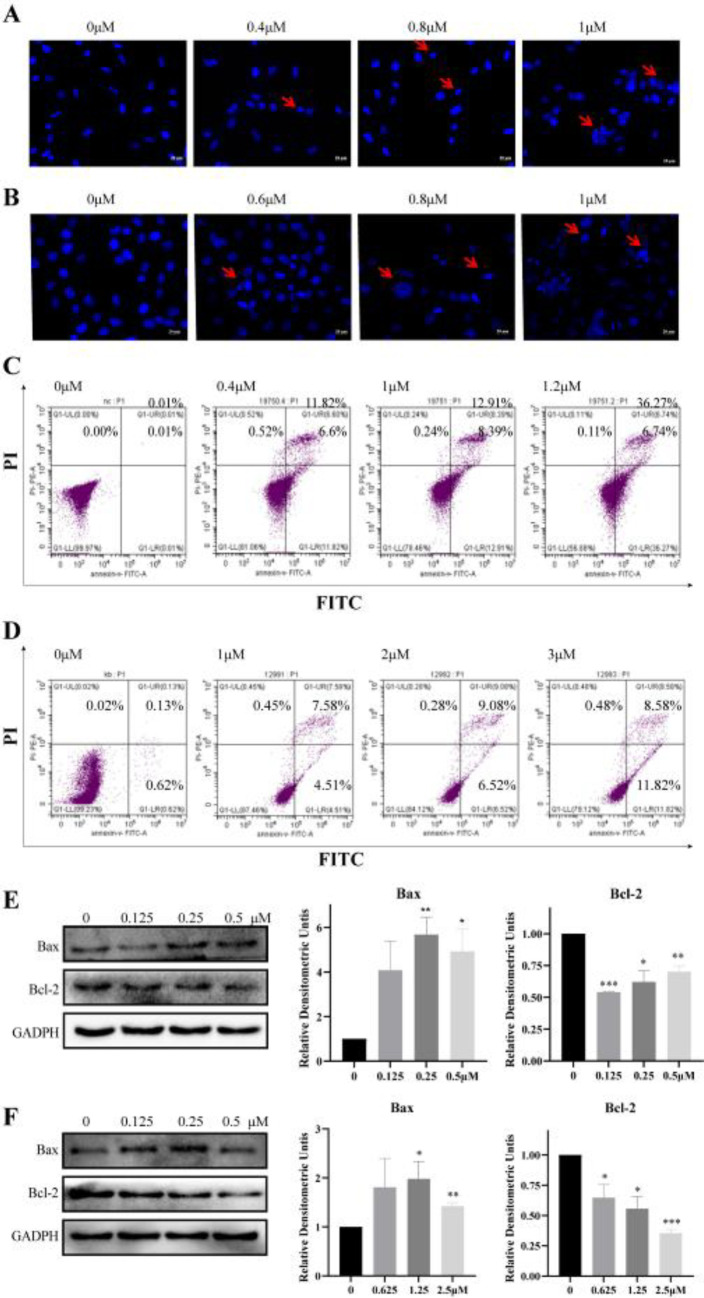
Sanguinarine induced apoptosis in H1975 and H1299 cells. (A) and (B) Morphological analysis with DAPI staining after 48 hr treatment of sanguinarine in H1975 and H1299 cells; (C) and (D) Apoptosis of H1975 and H1299 cells was analyzed by Annexin V-FITC/PI double staining and flow cytometry calculation; (E) and (F) Protein level of Bcl-2 and Bax in H1975 and H1299 cells treated with sanguinarine for 48 hr by Western blot. All experiments were carried out at least three times. *(*P*<0.05), **(*P*<0.01) were considered statistically significant compared with the controls

We further explored the effects of sanguinarine on cell apoptosis by DAPI staining. After 48 hr incubation with sanguinarine at indicated concentrations, cells became round and apoptotic bodies were observed under a fluorescence microscope (Figure 5A, B). The apoptotic analysis was also performed with Annexin V-FITC/PI double staining and evaluated by flow cytometry. H1975 and H1299 cells were pretreated with sanguinarine at gradient concentration for 48 hr, and then apoptosis cells were quantified. As shown in Figure 5C, D, the apoptotic cells were increased from 0.04% (0 μM, H1975), 0.75% (0μM, H1299) to 43.01% (1 μM, H1975, sanguinarine), and 20.4% (3μM, H1299, sanguinarine), respectively, indicating a dose-dependent effect of sanguinarine-induced apoptosis. To further clarify the mechanism of sanguinarine inducing apoptosis in lung cancer cells, we detected the markers of apoptosis, Bax, Bcl-2. Sanguinarine increased the pro-apoptosis protein Bax and decreased the anti-apoptosis protein Bcl-2 (Figure 5E, F). Therefore, we believe that sanguinarine may inhibit the growth and proliferation of lung cancer cells by inducing apoptosis.

***Sanguinarine reverses EMT process in NSCLC cell lines***

**Figure 6:**
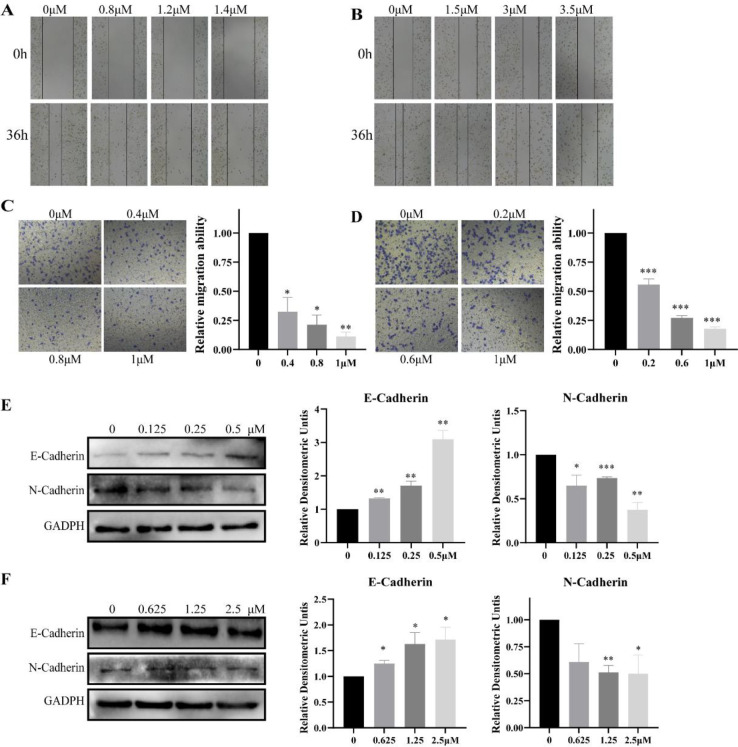
Effects of sanguinarine on cell migration, invasion in H1975 and H1299 cells. (A) and (B) Wound healing assay of H1975 and H1299 treated with different concentrations of sanguinarine for 36 hours; (C) and (D) Inhibition of H1975 and H1299 migration by sanguinarine; (E) and (F) Sanguinarine reverses the expression of E-Cadherin and N-Cadherin in NSCLC cell line H1975 and H1299, respectively. *(*P*< 0.05), **(*P*< 0.01) vs control. All experiments were carried out at least three times

It is well known that high expression of LSD1 can promote the EMT process (5) and induce migration and invasion in lung cancer cells (9, 34). We examined the effect of sanguinarine on the migratory capacity of lung cancer cells through wound healing and transwell experiments. H1975 and H1299 cells were treated with sanguinarine for 48 hr, microphotographs showed that the wound healing ability was significantly inhibited in a dose-dependent manner (Figures 6A, B). Moreover, sanguinarine also significantly inhibited the migration of H1975 and H1299 cells in a dose-dependent manner (Figure 6C, D). Subsequently, we detected the protein expression levels of EMT marker E-cadherin and N-cadherin in the two cell lines after 48 hr of drug treatment. Sanguinarine can significantly induce expression of E-Cadherin, an epithelial cell marker, while it can also suppress the expression of N-Cadherin, a mesenchymal cell marker (Figures 6E, F). In conclusion, sanguinarine can inhibit the migration and invasion of H1975 and H1299 cells, and promote the EMT process.

LSD1: Lysine-specific demethylase1

LSD1: Lysine-specific demethylase1

NSCLC: Non-small cell lung cancer

## Discussion

Natural products generally belong to biologically relevant chemical spaces with novel biological activities, making them rich sources of main compounds for the discovery of new drugs. As reported, nearly half of the small-molecular anticancer agents approved from the 1940s to 2012 are natural products or natural product derivatives (35). In recent years, several natural products have also shown promise in epigenetic drug discovery, some of which have entered clinical trials or are being used in clinics. For instance, romidepsin, a select class I and II histone deacetylase (HDAC) inhibitor, extracted from *Chromobacterium violaceum* and was approved for treating cutaneous T-cell lymphoma (36). Compelling evidence has indicated that dysregulation of various epigenetic pathways can contribute to cancer initiation and tumorigenesis. Among the epigenetic targets, LSD1 has diverse roles in physiological and pathological conditions, such as infections, immune modulation, and cancers, including lung cancer (37-39). Inactivation of LSD1 by RNAi or various kinds of inhibitors has been shown to effectively treat cancers. Thus, Targeting LSD1 is a promising option for cancer therapy. However, tranylcypromine-based irreversible LSD1 inhibitors in clinical trials have shown hematological toxicities, probably through producing FAD-adducts and disruption of the interaction between LSD1 and cofactors (40). To investigate whether non-covalent LSD1 inhibitors can improve their safety and efficacy, research on reversible LSD1 inhibitors with diverse structures is in full swing by many researchers. Until now, only two reversible LSD1 inhibitors CC-90011 and SP-2577 entered the clinical trials for treatment of advanced cancers and extensive-stage SCLC alone or combined with other antitumor drugs (41). Although the clinical phenotypes have not been fully defined and the side effects need to be further explored, novel reversible LSD1 inhibitors with diverse chemical structures and significant bioactivities would be a better choice since they may provide a safer metabolic profile. 

In this study, sanguinarine, a natural compound derived from *Macleaya cordata*, was identified as an LSD1 inhibitor for the first time by a fluorescence-based method. Further biochemical analysis revealed that sanguinarine is a reversible LSD1 inhibitor and LSD1 activity could be restored after dilution. According to a previous report, we also noted that several natural protoberberine alkaloids, which have a similar isoquinoline-based tetracyclic scaffold as sanguinarine, exhibited inhibitory activity against LSD1. Among them, epiberberine showed a better potency toward LSD1(IC_50_ = 0.14 μM) than sanguinarine (31). Given the structural similarity of sanguinarine with epiberberine, we compared their binding modes in the FAD pocket. Docking analysis indicated they occupied different positions in the FAD pocket, which suggested the importance of substituents in the activity. The structure-activity relationship studies will provide guidance for further modifications based on tetracyclic alkaloids. In addition, sanguinarine could also induce accumulation of H3K4me2/H3K9me2 and CD86 in the lung cancer cells and suppress cell growth. In NSCLC cell lines H1299 and H1975, sanguinarine significantly inhibited colony formation and induced morphological change concentration-dependently. Furthermore, sanguinarine could increase the expression of the key apoptosis proteins and induce H1299 and H1975 cell apoptosis. Meanwhile, Wound healing and transwell assays showed that sanguinarine inhibited the migration of H1975 and H1299 cells. EMT program is a central driver of tumor malignancy and serves as a major mechanism to promote cancer metastasis and invasion. During the transition, epithelial phenotypic marker E-cadherin will be lost, while mesenchymal phenotypic marker N-cadherin is elevated. It is reported that LSD1 can form a complex with snai1 to act at the E-Box domain of E-Cadherin and restrain the expression of E-cadherin for promoting cell migration and invasion (42, 29). Thus, inhibition of LSD1 activity may attenuate cell migration. In our study, sanguinarine increased the expression of E-cadherin and decreased the expression of N-cadherin in H1299 and H1975 cells. All results in this study demonstrate that sanguinarine is a cellular active LSD1 inhibitor, which sheds light on the search for more effective LSD1 inhibitors.

Although natural alkaloids have multiple biological activities, such as anti-inflammatory, antitumor, antimicrobial, and insecticidal effects, some adverse effects have also been reported. We found that sanguinarine exhibited obvious cytotoxicity against NSCLC cells, and was also weakly toxic to normal lung cell line LL24, but lung cancer cells were more sensitive to the antiproliferative effect of sanguinarine than the normal cells (43). The sanguinarine-mediated toxicity of Na^+-^K^+-^ATPase is responsible for abnormal cellular functions. Recent reports indicate that taking high doses of sanguinarine may cause DNA damage, however, the cytotoxicity and DNA damaging effect of sanguinarine are more specific to cancer cells than to normal cells (44, 45). In terms of effectiveness and toxicity, more studies are required especially with a focus on dosage. Furthermore, we believe that further structure optimization based on the tetracyclic scaffold on sanguinarine is a key way to obtain high efficiency and low toxicity candidate drug molecules.

## Conclusion

In summary, we screened a natural chemical library toward LSD1 and identified a potent, reversible LSD1 inhibition, sanguinarine, which displayed obvious anti-proliferative activity on all four NSCLC cell lines. Meanwhile, sanguinarine can promote the apoptosis of NSCLC cells and reverse the EMT process, and inhibit the proliferation and migration of NSCLC cells. Our findings implied sanguinarine has great potential for further optimization as a high-efficiency and low-toxicity inhibitor of LSD1. Besides, these findings give the potential application of sanguinarine in NSCLC with high expression of LSD1 and provide a novel scaffold for developing new LSD1 inhibitors. 

## Authors’ Contributions

TTQ, JGY, LXL Performed experimental investigation and analysis; XXW and KD Performed validation; TTQ Helped write the original draft; JLM and ZHL Helped write, review, and edit; JLM, ZHL, ZQZ Did funding acquisition. All authors have read and agreed to the published version of the manuscript.

## Conflicts of Interest

The authors declare that there are no conflicts of interest associated with this manuscript.
